# Artificial-intelligence-based MRI brain volumetry in patients with essential tremor and tremor-dominant Parkinson’s disease

**DOI:** 10.1093/braincomms/fcad271

**Published:** 2023-10-13

**Authors:** Veronika Purrer, Emily Pohl, Julia M Lueckel, Valeri Borger, Malte Sauer, Alexander Radbruch, Ullrich Wüllner, Frederic Carsten Schmeel

**Affiliations:** Department of Neurology, University Hospital Bonn, 53127 Bonn, Germany; German Center of Neurodegenerative Diseases (DZNE), 53127 Bonn, Germany; Department of Neurology, University Hospital Bonn, 53127 Bonn, Germany; Department of Diagnostic and Interventional Radiology, University Hospital Bonn, 53127 Bonn, Germany; Department of Neurosurgery, University Hospital Bonn, 53127 Bonn, Germany; Department of Neuroradiology, University Hospital Bonn, 53127 Bonn, Germany; German Center of Neurodegenerative Diseases (DZNE), 53127 Bonn, Germany; Department of Neuroradiology, University Hospital Bonn, 53127 Bonn, Germany; Department of Neurology, University Hospital Bonn, 53127 Bonn, Germany; German Center of Neurodegenerative Diseases (DZNE), 53127 Bonn, Germany; German Center of Neurodegenerative Diseases (DZNE), 53127 Bonn, Germany; Department of Neuroradiology, University Hospital Bonn, 53127 Bonn, Germany

**Keywords:** tremor, Parkinson’s disease, artificial intelligence, brain volumetry

## Abstract

Essential tremor and Parkinson’s disease patients may present with various tremor types. Overlapping tremor features can be challenging to diagnosis and misdiagnosis is common. Although underlying neurodegenerative mechanisms are suggested, neuroimaging studies arrived at controversial results and often the different tremor types were not considered. We investigated whether different tremor types displayed distinct structural brain features. Structural MRI of 61 patients with essential tremor and 29 with tremor-dominant Parkinson’s disease was analysed using a fully automated artificial-intelligence-based brain volumetry to compare volumes of several cortical and subcortical regions. Furthermore, essential tremor subgroups with and without rest tremor or more pronounced postural and kinetic tremor were investigated. Deviations from an internal reference collective of age- and sex-adjusted healthy controls and volumetric differences between groups were examined; regression analysis was used to determine the contribution of disease-related factors on volumetric measurements. Compared with healthy controls, essential tremor and tremor-dominant Parkinson’s disease patients displayed deviations in the occipital lobes, hippocampus, putamen, pallidum and mesencephalon while essential tremor patients exhibited decreased volumes within the nucleus caudatus and thalamus. Analysis of covariance revealed similar volumetric patterns in both diseases. Essential tremor patients without rest tremor showed a significant atrophy within the thalamus compared to tremor-dominant Parkinson’s disease and atrophy of the mesencephalon and putamen were found in both subgroups compared to essential tremor with rest tremor. Disease-related factors contribute to volumes of occipital lobes in both diseases and to volumes of temporal lobes in essential tremor and the putamen in Parkinson’s disease. Fully automated artificial-intelligence-based volumetry provides a fast and rater-independent method to investigate brain volumes in different neurological disorders and allows comparisons with an internal reference collective. Our results indicate that essential tremor and tremor-dominant Parkinson’s disease share structural changes, indicative of neurodegenerative mechanisms, particularly of the basal-ganglia-thalamocortical circuitry. A discriminating, possibly disease-specific involvement of the thalamus was found in essential tremor patients without rest tremor and the mesencephalon and putamen in tremor-dominant Parkinson’s disease and essential tremor without rest tremor.

## Teaser

Using an artificial-intelligence-based volumetry in essential tremor and tremor-dominant Parkinson’s disease, Purrer *et al*. investigated structural brain features in different tremor types. They found similar volumetric patterns in both diseases but significant deviations from a normative database, particularly within the basal-ganglia-thalamocortical circuitry, suggesting that both diseases share structural changes, indicative of neurodegenerative mechanisms.

## Graphical Abstract

**Graphical abstract:**
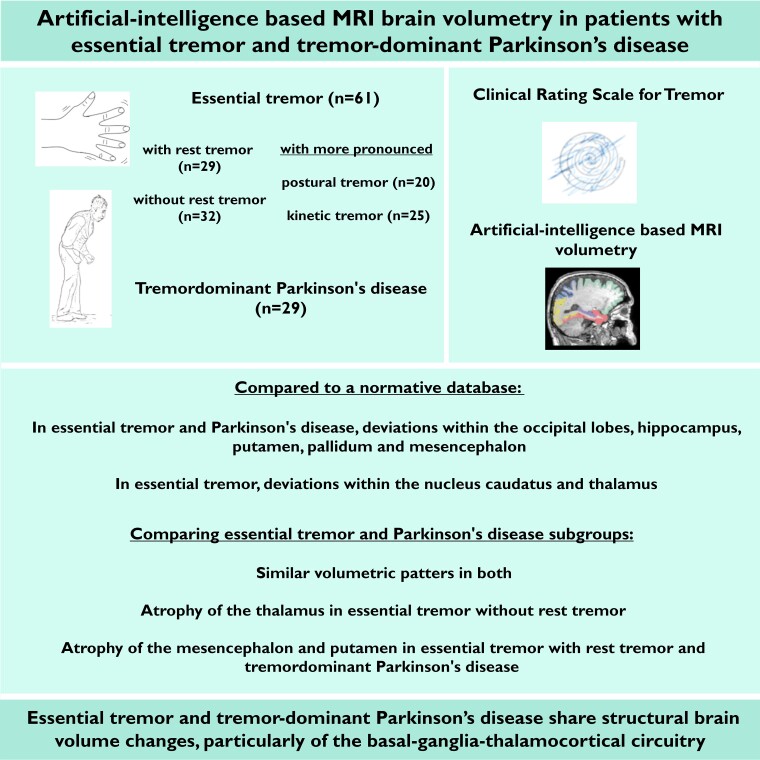

## Introduction

Tremor is a common neurological disorder and often leads to significant impairments in everyday life. Essential tremor (ET) and tremor of Parkinson’s disease, which is among the most common forms of tremor, differ traditionally in different clinical features, such as body distribution or activation condition of the tremor. Classic ET is characterized by bilateral action tremor of the upper extremities. However, additional clinical symptoms, particularly cerebellar signs such as intention tremor, rest tremor or impaired tandem gait and involvement of other body parts are common. Tremor in Parkinson’s disease typically is an asymmetric tremor at rest but may also be accompanied by a mostly less pronounced action tremor. Clinical differentiation of ET and Parkinson’s disease can be challenging due to the significant overlap of symptoms, potentially leading to misdiagnosis of the underlying aetiology.^1,2^ Moreover, evidence of overlapping motor and non-motor symptoms and an increased prevalence of (particularly tremor-dominant (td)) Parkinson’s disease in patients with previous ET or their relatives, or the co-occurrence of both diseases, suggest an association between ET and Parkinson’s disease.^3^

Although the pathophysiology of both diseases is not fully understood, recent evidence implicates underlying neurodegenerative mechanisms. In addition to clinical patterns, pathological and neuroimaging findings also point to certain common pathophysiological features of ET and Parkinson’s disease.^4^ Previous studies identified increased activity within the cerebello-thalamocortical circuit in both, ET and Parkinson’s disease although. However, the underlying pathophysiological mechanisms are considered to be different. In ET, GABAergic dysfunction of the dentate nucleus and degeneration of the brainstem and cerebellum are thought to drive tremor activity. In contrast, there is evidence that pallidal dopamine deficiency is the likely cause in Parkinson’s disease.^4^ Nevertheless, the fact that the basal ganglia and cerebellar circuits are anatomically connected and that stereotactic treatments, such as deep brain stimulation (DBS) of the posterior subthalamic area, can improve tremor in both diseases raises the possibility that ET and Parkinson’s disease share certain pathophysiological features.^5-7^

Previous imaging studies investigated ET and Parkinson’s disease by voxel-based morphometry (VBM) and manual volume segmentation, with different studies hitherto arriving at divergent conclusions about abnormalities in cortical and subcortical regions. Although atrophy of the cerebellum, basal ganglia, thalamus, parietal lobes, frontal lobes, occipital lobes, insula, or temporal lobes has been described in both diseases, the results have not yet been sufficiently reproduced across studies.^8-13^ Furthermore, most previous studies on Parkinson’s disease did not address the different clinical subtypes, notably td Parkinson’s disease. To date, few studies have compared volumetric measurements in ET and Parkinson’s disease patients, and given the different study designs as well as variations in study cohorts and mostly manual MRI evaluation methods, comparability of results remains limited.^14-17^ Therefore, it is still uncertain whether a more reliable measurement of potentially affected brain regions is possible using fully automated, quantitative software-based and thus, objective MRI evaluation methods. Automated segmentation methods could provide a rapid, investigator-independent, and highly reproducible tool for segmenting cortical and subcortical structures and have been shown to be an efficient technique in volumetric studies of ET and Parkinson’s disease.^17,18^ Based on these considerations, the aim of our study was to (i) detect potential structural brain changes in patients with ET and td Parkinson’s disease using automated artificial-intelligence (AI)-based brain MRI volumetry and (ii) test the discriminatory value of the detected brain volume changes for distinguishing ET and Parkinson’s disease patients.

## Material and methods

### Study cohort

Between October 2018 and October 2022, 61 patients with a clinical diagnosis of ET and 29 patients with td Parkinson’s disease were recruited in the outpatient department for movement disorders at University Hospital Bonn. Patients presented for consideration of transcranial high-intensity Magnetic Resonance-guided Focused Ultrasound (MRgFUS) treatment, and therefore all patients had a moderate to severe, medication-refractory tremor (score of ≥ 2 in the dominant hand on the Clinical Rating Scale for Tremor (CRST). Diagnosis of ET or Parkinson’s disease was confirmed by neurologists specialized in movement disorders according to the International Parkinson disease and Movement Disorder Society consensus criteria.^19,20^ All patients with Parkinson’s disease showed a tremor-dominant subtype classified based on established methods calculating a tremor/non-tremor score.^21^ Using items from the Movement Disorder Society-Unified Parkinson’s Disease Rating Scale (MDS-UPDRS) III,^22^ we calculated the tremor score (sum of item 3.15–3.17 divided by 9) and non-tremor score (sum of item 3.1–3.3, 3.9–3.10, 3.12–3.14 divided by 12). A tremor-dominant subtype was defined by a tremor/non-tremor ratio equal or greater than 1. Clinical testing and MRI scans were conducted directly before MRgFUS treatment and for this purpose the current medications had to be stable for at least 30 days at the time point of enrolment and had to be discontinued before treatment (1 week prior in ET and at least 12 hours overnight in Parkinson’s disease).

The study was performed according to the Declaration of Helsinki and approved by the local Ethics Committee (314/18). All participants provided written, informed consent.

### Clinical evaluation

All patients underwent a careful clinical evaluation (conducted by two neurologists with 30 and 6 years of experience in movement disorders). For tremor assessment the CRST^23^ was used, the MDS-UPDRS III was assessed additionally in Parkinson’s disease patients. On both scales, laterality-specific (right/left), affection-specific (more/less severely affected) and condition-specific (rest/postural/kinetic) subscales were created. Higher values indicate more severe tremor. Furthermore, we created subgroups of patients with ET with rest tremor (ET_R_) and without rest tremor (ET_WR_) or a more pronounced postural (ET_P_) or kinetic (ET_K_) tremor.

### Image acquisition and analysis

Standardized MR imaging was acquired at a clinical 3T scanner (Philips Achieva TX, Best, The Netherlands) equipped with an eight-channel head coil. The protocol comprised a 3D T1-weighted magnetization-prepared rapid gradient-echo (MPRAGE) sequence acquired with an isotropic resolution of 1 mm and the following parameters: repetition time (TR) = 7.286 ms, echo time (TE) = 3.93 ms, matrix = 256 × 256 mm, 180 slices in total, scan time = 4.66 min. Automated AI-based software was used to determine quantitative analyses of the volume of different brain areas in ml and age- and sex-adjusted percentiles (reported as deviation of two or four standard deviations based on an internal reference collective of age- and sex-adjusted healthy controls (HC) from the general population embedded in the software). This commercially licenced MRI post-processing software named ‘mdbrain’ is provided by Mediaire GmbH, Berlin, Germany, which is an approved medical device manufacturer according to the European Medical Device Directive 93/42/EEC and is certified according to DIN EN ISO 13485:2016. The ‘mdbrain’ software is approved as a Conformité Européenne (CE)-marked medical device and performs automatic brain volumetry of different brain regions using native 3D T1 weighted sequences to allow quantitative statements based on an extensive population-based normative database. The system leverages a custom deep learning segmentation model based on the U-Net architecture to perform a highly accurate side- and region-specific (e.g. lobes) rapid brain volumetry, which was trained on a heterogeneous dataset of 3D T1w images (*n* = 2869, balanced m/f). Augmentation techniques (augmentation of contrast, resolution, rotation, elastic deformation) have been used to maximize the model’s applicability in daily routine. The volumes of 42 brain regions including the hippocampus are determined and percentiles are derived by comparison to a cohort of healthy individuals (*n* = 6371, balanced m/f, age range 10–97) while accounting for age, sex and total intracranial volume (ICV). The following volumes were extracted: total brain volume (TBV), grey matter (GM) and white matter (WM), cortical GM (cGM), brainstem (as well as mesencephalon and pons separately), cerebellar cortex and all ventricles. Furthermore, several cortical and subcortical areas were assessed bilaterally as well as for each side separately: frontal, parietal (as well as the precuneus separately), occipital, temporal lobe (as well as the hippocampus, parahippocampal gyrus and entorhinal cortex separately), nucleus caudatus, putamen, pallidum and thalamus (Supplementary Fig. 1).

### Statistical analysis

Statistical analysis was carried out using IBM SPSS Statistics for Windows, version 25 (IBM Corp., Armonk, NY, USA). Shapiro–Wilk test was used to evaluate the normal distribution of the data. Since most of the volumes were normally distributed, parametric tests were used for further analysis. Group differences in demographical and clinical data were evaluated with the χ^2^ test (sex and handedness) and independent sample *t*-test (age, age of onset, disease duration, total CRST scale and subscales). Differences within both groups were examined using the paired *t*-test (CRST and MDS-UPDRS subscales).

Group differences in the incidence of deviations of single brain volumes from the norm collective were analysed using the χ^2^ test and Bonferroni’s correction for multiple comparisons. Instead of right–left, supratentorial, side-specific volumes were arranged according to the more-less affected side (contralateral to the more/less affected extremity).

For volumetric analysis, all acquired brain volumes were normalized to the ICV of each subject. To determine the estimated ICV, the TBV and total ventricle volume were summed up. Volumetric measurements were assessed between the groups using the analysis of covariance (ANCOVA) and adjusting for age and sex. In the case of more than two groups (ET_R_, ET_-R_, td Parkinson’s disease) a *post hoc* Bonferroni test was applied. In both groups, a hierarchical multiple linear regression was used to determine the contribution of disease duration and tremor severity (clinical scores) on volumetric measurements while controlled for age and sex. Therefore, age and sex were entered in the first block. Next, disease duration and the CRST score were added as predictors.

*P*-values <0.05 were considered statistically significant. Effect sizes were interpreted according to Cohen.^24^ Continuous variables are presented as mean ± standard deviation (SD) and categorical variables as frequency and percentage.

## Results

### Demographical and clinical characteristics

The mean age in ET (69.9 ± 12.9 years) and Parkinson’s disease (64.1 ± 10.8 years) patients differed slightly (*P* = 0.04). No statistically significant difference was found in the other demographic characteristics (i.e. sex and handedness). ET patients reported a younger age of onset and more prolonged disease duration. Total CRST score and subscores were higher in ET patients. In both groups separately, the side-specific subscores did not differ significantly (ET: *P* = 0.88; Parkinson’s disease: *P* = 0.12) but comparing the more to the less severely affected side we found a significant aberration in both groups, which was higher in Parkinson’s disease patients (ET: mean difference (MD) = 3.1, *P* < 0.001; Parkinson’s disease: MD = 9.4, *P* < 0.001). Comparing the more to the less severely affected side on the MDS-UPDRS scale, asymmetric tremor was found in Parkinson’s disease patients (MD = 5.9, *P* < 0.001). The mean tremor/non-tremor ratio for Parkinson’s disease patients was 2.0 ± 1.9. Furthermore, Parkinson’s disease patients showed a more pronounced rest tremor, while postural and kinetic tremor was more evident in ET patients. There were no significant demographic differences between ET_R_ and ET_WR_ as well as between ET_P_ and ET_K_ but tremor scores were higher in ET_R_ and ET_K_ compared to ET_WR and_ ET_P_, respectively. Detailed demographical and clinical characteristics are given in Table 1.

**Table 1:** Demographic and clinical characteristics of the study participants (*n* = 90)

	ET (*n* = 61)		Parkinson’s disease (*n* = 29)		*P*-value^g^	
Age—yr^a^	69.9 ± 12.9		64.1 ± 10.8		0.04	
Male sex—no. (%)	45 (74%)		25 (86%)		0.19	
Right-handedness—no. (%)	57 (93%)		27 (93%)		0.95	
Age of onset^a^	41.4 ± 20.8		58.3 ± 11.1		<0.001	
Disease duration^a^	28.3 ± 16.5		5.8 ± 2.7		<0.001	
CRST^a^						
Total score^b^	59.0 ± 16.2		32.6 ± 15.3		<0.001	
CRST_right_^c^	18.9 ± 5.5		12.6 ± 8.3		0.001	
CRST_left_^c^	18.9 ± 5.7		9.3 ± 5.7		<0.001	
CRST_moreaff_^c^	20.5 ± 5.1		15.7 ± 6.0		<0.001	
CRST_lessafft_^c^	17.3 ± 5.7		6.3 ± 5.1		<0.001	
CRST_rest_^c^	1.1 ± 1.4		5.3 ± 1.0		<0.001	
CRST_postural_^c^	6.5 ± 2.0		3.9 ± 1.9		<0.001	
CRST_kinetic_^c^	6.9 ± 2.8		2.0 ± 1.7		<0.001	
UPDRS-III^a^						
Total score^d^			35.7 ± 13.9			
UPDRS_right_^e^			14.1 ± 6.8			
UPDRS_left_^e^			10.5 ± 7.6			
UPDRS_tremor_right_^f^			6.7 ± 3.4			
UPDRS_tremor_left_^f^			4.0 ± 3.2			
UPDRS_tremor_moreaff_^f^			8.3 ± 1.6			
UPDRS_tremor_lessaff_^f^			2.4 ± 2.2			
	ET_R_ (*n* = 29)	ET_WR_ (*n* = 32)	*P*-value^g^	ET_P_ (*n* = 20)	ET_K_ (*n* = 25)	*P*-value^g^
Age—yr^a^	70.5 ± 14.4	69.3 ± 11.7	0.71	68.0 ± 15.7	69.0 ± 10.9	0.80
Male sex—no. (%)	21 (72%)	24 (75%)	0.40	15 (75%)	17 (68%)	0.84
Right-handedness—no. (%)	28 (97%)	29 (91%)	0.65	18 (90%)	25 (100%)	0.28
Age of onset^a^	42.6 ± 21.2	40.3 ± 20.7	0.66	39.2 ± 20.9	40.1 ± 21.9	0.89
Disease duration^a^	27.4 ± 16.1	29.0 ± 17.0	0.71	28.8 ± 17.6	28.9 ± 17.6	0.98
CRST^a^						
Total score^b^	64.0 ± 16.9	54.5 ± 14.3	0.025	52.0 ± 15.0	64.8 ± 13.5	0.004
CRST_right_^c^	20.1 ± 5.5	17.9 ± 5.3	0.12	16.9 ± 5.4	20.8 ± 4.5	0.012
CRST_left_^c^	20.6 ± 5.5	17.3 ± 5.5	0.025	16.4 ± 5.2	20.9 ± 5.1	0.005
CRST_moreaff_^c^	21.9 ± 5.0	19.2 ± 4.9	0.05	18.3 ± 4.9	22.4 ± 4.2	0.004
CRST_lessafft_^c^	18.9 ± 5.6	15.9 ± 5.4	0.042	15.0 ± 5.1	19.3 ± 4.9	0.006
CRST_rest_^c^	2.3 ± 1.2	0	<0.001	1.1 ± 1.3	1.2 ± 1.5	0.89
CRST_postural_^c^	7.2 ± 1.8	6.0 ± 2.1	0.023	6.4 ± 2.0	6.4 ± 2.0	1.0
CRST_kinetic_^c^	7.4 ± 2.7	6.4 ± 2.8	0.17	4.6 ± 1.8	8.7 ± 2.3	<0.001

^a^Values are means ± SD.

^b^Consisting of subscores A (clinical observation), B (motor tasks) and C (subjective disability). The total score ranges from 0 to 144.

^c^Right/left = modified score of the clinical examination and motor tasks of the right or left upper and lower extremity. Subscores ranges from 0 to 40 each. More/less affected = flipped side-specific subscores according to higher or lower values. Subscores ranges from 0 to 40 each. Rest/postural/kinetic = modified score of the clinical examination of the right and left upper and lower extremity in rest, postural or kinetic condition. Subscores ranges from 0 to 16 each.

^d^The total score ranges from 0 to 132.

^e^Right/left = modified score of the clinical examination of the right or left upper and lower extremity. Subscores ranges from 0 to 44 each.

^f^Tremor_right/left = modified tremor score of the clinical examination of the right or left upper and lower extremity. Subscores ranges from 0 to 16 each. Moreaff/lessaff = flipped side-specific subscores according to higher or lower values. Subscores ranges from 0 to 16 each.

^g^A *P*-value < 0.05 was considered statistically significant. Group differences were calculated using Pearson-Chi-Quadrat and the independent sample *t*-test.

Abbreviations: ET, essential tremor; CRST, Clinical Rating Scale for Tremor; UPDRS, MDS-Unified Parkinson’s Disease Rating Scale; ET_R_, essential tremor with rest tremor; ET_WR,_ essential tremor without rest tremor; ET_P,_ essential tremor with pronounced postural tremor; ET_K,_ essential tremor with pronounced kinetic tremor.

### Brain volume deviations in ET and Parkinson’s disease as compared to a norm collective

**Figure 1:**
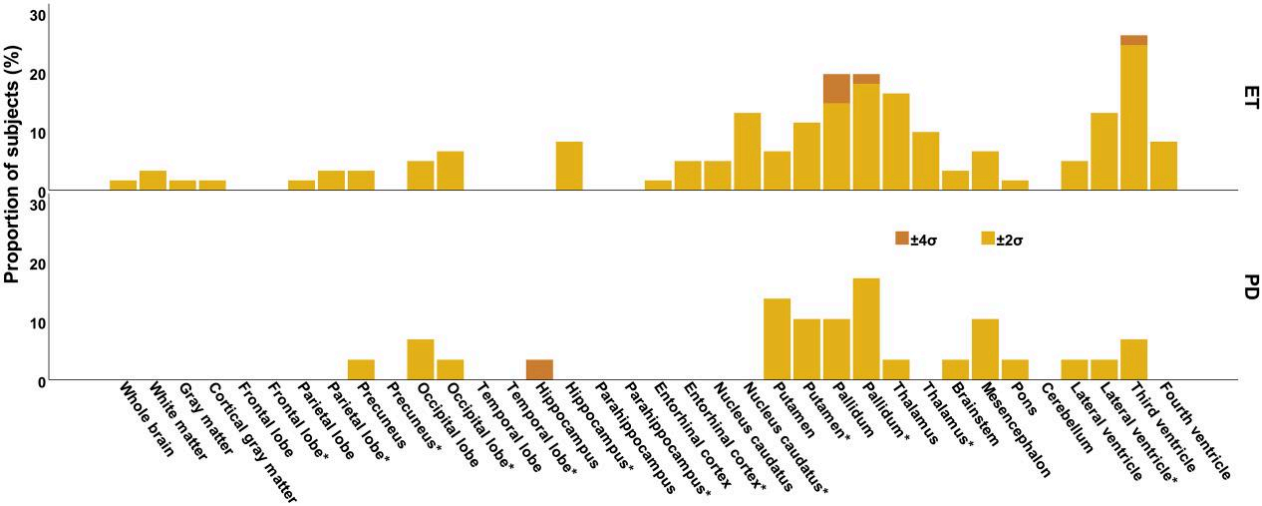
**Percentage proportion of subjects with volume deviations (%).** Incidence of deviations [two or four standard deviations] of the single brain volumes compared with a norm collective. Deviations of supratentorial volumes are provided separately for each side only (the volume contralateral to the clinically more affected side has been indicated with *). Deviations of cortical and subcortical volumes correspond to atrophy, deviations of ventricular volumes to enlargement. Significant deviations were found within the nucleus caudatus contralateral to the clinically more affected side and the thalamus contralateral to the clinically less affected side.

Comparing the individual brain (sub)volumes of patients with ET and td Parkinson’s disease with the embedded population-based norm collective, a significant proportion of subjects with volume deviations from the norm collective was found especially in the occipital lobes, hippocampus, nucleus caudatus, putamen, pallidum, thalamus and mesencephalon (Fig. 1, Supplementary Table 1). In ET, a significantly greater proportion of subjects with volume deviations from the norm collective compared to Parkinson’s disease were found within the nucleus caudatus contralateral to the clinically more affected side (*P* = 0.04). Within the thalamus contralateral to the clinically less affected sides significantly greater proportion was evident in the ET_WR_ group compared to ET_R_ and Parkinson’s disease (*P* < 0.001 and *P* = 0.008; a *P*-value <0.017 was considered as statistically significant after Bonferroni correction). In ET_K_ and ET_P_ the incidence of deviations from the norm collective did not differ.

### Volumetric results in ET and Parkinson’s disease patients

In ET and Parkinson’s disease patients, relative volumetric measurements of different brain structures overlapped (Table 2, Supplementary Table 2). Compared to ET patients, the TBV in Parkinson’s disease was only slightly increased (+1%). The largest increase was found in the occipital lobe ipsilateral (+3%) and the thalamus contralateral to the more affected extremity (+3%), whereas the largest brain volume loss was evident in the nucleus caudatus contralateral (−5%) and putamen ipsilateral to the more affected side (−5%) as well as a large ventricle volume loss (−26%) compared to ET. Interestingly, the cerebellum showed a volume loss of 2% in Parkinson’s disease patients compared to ET, even when Parkinson’s disease patients were compared to ET patients with pronounced kinetic tremor. Significant volumetric differences were detected only in the frontal lobe contralateral to the less affected side (F(1,85) = 3.99, *P* = 0.049, η² = 0.045), but the effect size was weak (f = 0.22).

**Table 2:** Results of volumetric MRI analysis in ET and Parkinson’s disease patients

	ET (*n* = 61)		Parkinson’s disease (*n* = 29)			
	Mean	SD	Mean	SD	% Δ	*P*-value^a^
ICV	1.000		1.000			
TBV	0.963	0.0188	0.972	0.0139	1	0.534
WM	0.422	0.0170	0.431	0.0197	2	0.102
GM	0.541	0.0178	0.541	0.0174	0	0.180
Cortical GM	0.369	0.0146	0.373	0.0155	1	0.320
Frontal lobe^c^						
Total	0.133	0.0075	0.136	0.0072	2	0.071
More affected	0.066	0.0039	0.068	0.0033	2	0.161
Less affected	0.067	0.0038	0.069	0.0042	2	0.049 ^b^
Parietal lobe^c^						
Total	0.073	0.0046	0.074	0.0044	1	0.634
More affected	0.037	0.0024	0.037	0.0023	1	0.490
Less affected	0.036	0.0028	0.037	0.0024	1	0.871
Occipital lobe^c^						
Total	0.053	0.0033	0.055	0.0034	3	0.078
More affected	0.027	0.0021	0.028	0.0021	2	0.889
Less affected	0.026	0.0023	0.027	0.0025	3	0.480
Temporal lobe^c^						
Total	0.109	0.0055	0.109	0.0052	−1	0.765
More affected	0.054	0.0033	0.054	0.0033	0	0.882
Less affected	0.055	0.0033	0.055	0.0027	−1	0.713
Caudate^c^						
Total	0.005	0.0009	0.005	0.0007	−3	0.872
More affected	0.003	0.0005	0.002	0.0003	−5	0.464
Less affected	0.003	0.0005	0.003	0.0005	−2	0.687
Putamen^c^						
Total	0.007	0.0007	0.006	0.0005	−4	0.309
More affected	0.003	0.0004	0.003	0.0003	−3	0.241
Less affected	0.003	0.0004	0.003	0.0003	−5	0.428
Pallidum^c^						
Total	0.002	0.0002	0.002	0.0002	1	0.133
More affected	0.001	0.0001	0.001	0.0001	1	0.144
Less affected	0.001	0.0001	0.001	0.0001	2	0.164
Thalamus^c^						
Total	0.012	0.0008	0.013	0.0007	2	0.857
More affected	0.006	0.0005	0.006	0.0004	3	0.830
Less affected	0.006	0.0005	0.006	0.0004	2	0.602
Brainstem	0.022	0.0016	0.022	0.0015	−1	0.396
Mesencephalon	0.006	0.0004	0.006	0.0004	−2	0.287
Pons	0.012	0.0010	0.012	0.0010	0	0.639
Cerebellum	0.085	0.0067	0.083	0.0048	−2	0.565
Ventricle volume	0.037	0.0188	0.028	0.0139	−26	0.534

Relative volumes of the different brain volumes for ET and Parkinson’s disease patients. All volumes have been normalized to the ICV.

^a^p-value of the ANCOVA performed with age, sex, age of onset and disease duration as covariates.

^b^ *P*-value <0.005 was considered statistically significant.

^c^the more/less affected brain volumes is defined as the volume contralateral to the more/less affected tremor side.

Abbreviations: ET, essential tremor; SD, standard deviation; % Δ, percentage difference of the mean of Parkinson’s disease patients to the mean of ET patients; ICV, intracranial volume.

Comparing td Parkinson’s disease patients with the ET_WR_ group and the ET_R_ group revealed significant differences within the TBV (td Parkinson’s disease versus ET_WR_ MD = 0.012, *P* = 0.002; td Parkinson’s disease versus ET_R_ MD = 0.008, *P* = 0.049), hippocampus (ET_R_ versus ET_WR_ MD = 0.0003, *P* = 0.044), mesencephalon (td Parkinson’s disease versus ET_R_ MD = −0.0002, *P* = 0.041; ET_R_ versus ET_WR_ MD = 0.0002, *P* = 0.018), thalamus contralateral to the more affected side (td Parkinson’s disease versus ET_WR_ MD = 0.0002, *P* = 0.018), putamen contralateral to the more affected side (td Parkinson’s disease versus ET_R_ MD = −0.0002, *P* = 0.031; ET_R_ versus ET_WR_ MD = 0.0002, *P* = 0.044) and both lateral ventricles (contralateral to more affected side: td Parkinson’s disease versus ET_R_ MD = −0.0043, *P* = 0.045; td Parkinson’s disease versus ET_WR_ MD = −0.0058, *P* = 0.004; contralateral to less affected side: td Parkinson’s disease versus ET_WR_ MD = −0.0052, *P* = 0.003) (Supplementary Table 3). In addition, no differences were found between ET_P_ and ET_K_ (Supplementary Table 4).

**Figure 2:**
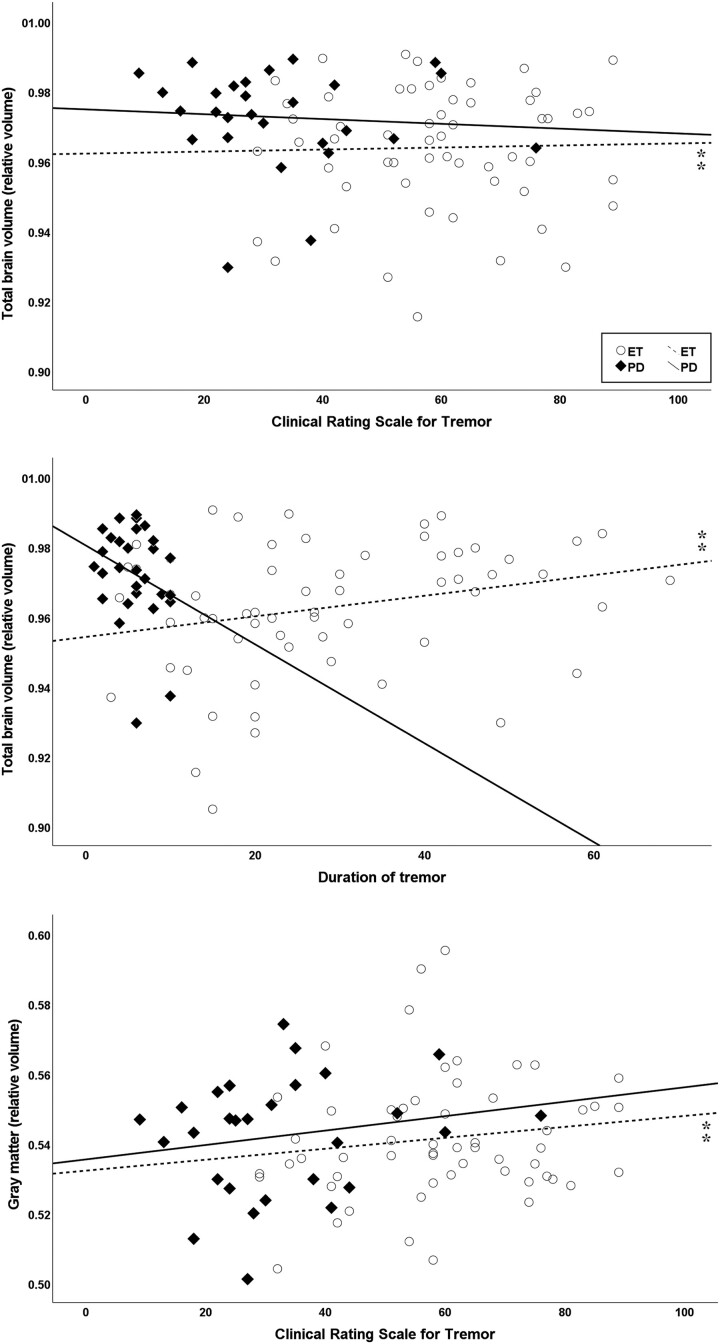
**Scatter plots showing volumes correlating with disease-related factors (disease duration or tremor severity score CRST) while controlled for age and sex using hierarchical multiple linear regression.** In ET, significant correlations (⁑) were found for the TBV (ΔF = 5.25, ΔR² = 0.09) with disease duration (*P* = 0.01) and CRST scores (*P* = 0.04). Furthermore, significant effects were evident for the GM (ΔF = 3.6, ΔR² = 0.08) with CRST score (*P* = 0.01). All volumes have been normalized to the ICV.

**Figure 3:**
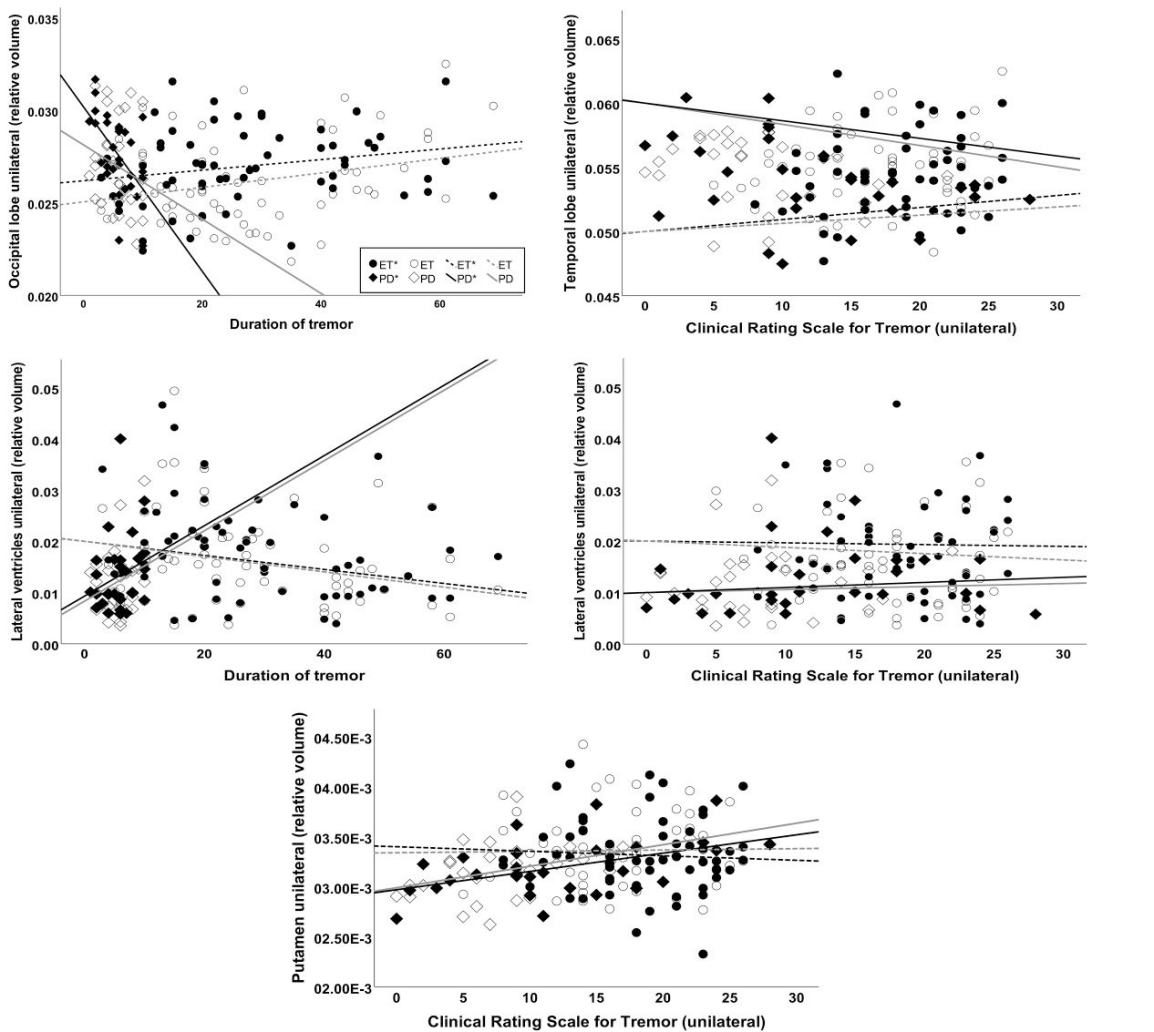
**Scatter plots showing volumes correlating with disease-related factors (disease duration or tremor severity score CRST) while controlled for age and sex using hierarchical multiple linear regression.** In ET, significant correlations (⁑) were found for the lateral ventricles (ΔF = 5.0, ΔR² = 0.09) with disease duration (*P* = 0.02) and CRST scores (*P* = 0.047). Furthermore, significant effects were evident for the temporal lobe (ΔF = 4.37, ΔR² = 0.13) with CRST score (*P* = 0.005) and the occipital lobe with disease duration (ΔF = 4.22, ΔR² = 0.13, *P* = 0.006). In td Parkinson’s disease, disease duration contributed to occipital lobe volume (ΔF = 4.03, ΔR² = 0.24, *P* = 0.01) and the CRST to the putaminal volume (ΔF = 6.84, ΔR² = 0.37, *P* = 0.005). The volume contralateral to the clinically more affected side has been indicated with *. All volumes have been normalized to the ICV.

Hierarchical linear regression controlled for sex and age was used to determine whether disease duration or clinical scores contributed to the volumetric measurements while controlling for sex and age. In ET patients, the model showed that age and sex were associated with several cortical and subcortical brain areas, whereas in td Parkinson’s disease patients, associations with age were found only in the volumes of the TBV, pallidum and lateral ventricles (Supplementary Table 5). After adjusting for demographics, we found a weak but statistically significant association of the TBV and lateral ventricles with disease duration and CRST score as well as of the GM with CRST score in ET patients. Furthermore, a moderate effect was evident for the occipital lobe and disease duration and the temporal lobe and CRST score. In td Parkinson’s disease, results of hierarchical regression indicated that disease duration contributed moderately to occipital lobe volume. A strong contribution was shown for CRST and the putamen (Table 3, Figs 2 and 3). After analysing the supratentorial volumes separately, weak associations of the entorhinal lobe contralateral to the less affected side and disease duration as well as of both ventricles and disease duration and CRST score were found in ET patients. In td Parkinson’s disease, moderate effects were observed between disease duration and the parietal lobe and occipital lobe contralateral to the more affected side as well as CRST score and both putamina (Supplementary Tables 6 and 7).

**Table 3:** Disease-related correlates of volumetric measurements in ET and Parkinson’s disease patients

	ET (*n* = 61)		Parkinson’s disease (*n* = 29)	
	DOT	CRST	DOT	CRST
TBV				
Std. β^a^	0.24*	0.21*	−0.29	−0.01
ΔR²	0.09		0.08	
ΔF	5.25**		2.14	
WM				
Std. β^a^	0.19	−0.09	0.11	−0.25
ΔR²	0.04		0.06	
ΔF	1.20		0.86	
GM				
Std. β^a^	0.06	0.29*	−0.36	0.28
ΔR²	0.08		0.15	
ΔF	3.60*		2.08	
Cortical GM				
Std. β^a^	0.08	0.20	−0.42	0.21
ΔR²	0.04		0.16	
ΔF	2.06		2.44	
Frontal lobe				
Std. β^a^	0.07	0.07	−0.35	0.40
ΔR²	0.01		0.20	
ΔF	0.52		2.96	
Parietal lobe				
Std. β^a^	−0.09	0.03	−0.35	0.09
ΔR²	0.01		0.11	
ΔF	0.36		1.64	
Occipital lobe				
Std. β^a^	0.36**	0.08	−0.50*	−0.02
ΔR²	0.13		0.24	
ΔF	4.22*		4.03*	
Temporal lobe				
Std. β^a^	−0.04	0.38**	−0.19	< −0.01
ΔR²	0.13		0.04	
ΔF	4.37*		0.44	
Caudate				
Std. β^a^	−0.16	−0.20	0.12	−0.01
ΔR²	0.06		0.01	
ΔF	1.87		0.15	
Putamen				
Std. β^a^	−0.06	−0.04	0.18	0.53**
ΔR²	0.01		0.37	
ΔF	0.14		6.84**	
Pallidum				
Std. β^a^	0.17	0.06	0.10	0.11
ΔR²	0.03		0.03	
ΔF	0.81		0.45	
Thalamus				
Std. β^a^	0.13	0.14	−0.09	−0.06
ΔR²	0.03		0.01	
ΔF	0.99		0.16	
Brainstem				
Std. β^a^	0.06	−0.02	0.03	0.20
ΔR²	<0.01		0.04	
ΔF	0.13		0.51	
Mesencephalon				
Std. β^a^	0.01	−0.08	0.15	0.01
ΔR²	0.01		0.02	
ΔF	0.16		0.30	
Pons				
Std. β^a^	0.08	−0.01	0.03	0.21
ΔR²	0.01		0.05	
ΔF	0.23		0.64	
Cerebellum				
Std. β^a^	0.01	0.23	−0.02	0.26
ΔR²	0.05		0.07	
ΔF	1.34		0.84	

Hierarchical multiple regression analyses for ET and Parkinson’s disease patients. Regression analyses consisted of two steps: (i) demographic variables (age and sex) were entered as the first block, and (ii) disease-related factors (DOT and tremor severity measured by the CRST) were entered in the second block. For results of the first block, we refer to the Supplementary Table 5.

** *P* < 0.01, * *P* < 0.05.

^a^All standarized regression coefficients are from the final step in the analyses.

Abbreviations: ET, Essential Tremor; DOT, Duration of tremor; CRST, Clinical Rating Scale for Tremor.

## Discussion

In this study, we used for the first time an automated AI-based MR imaging brain volumetry to compare possible structural differences in medication-refractory ET and td Parkinson’s disease patients while past studies mostly relied on manual or semi-automated segmentation methods. Manual segmentation is time-consuming, highly dependent on investigator experience and less comparable because of individual segmentation in the native space without image transformation. Fully automated methods can provide a fast, reliable tool for measuring the volumes of different cortical and subcortical brain regions. The software used in this study has been shown to be a reliable method for brain volumetry in various neurological diseases^25,26^ and received CE-mark approval for medical reporting in clinical practice. In contrast, in-house software solutions, which have been tailored mostly for research purposes are less suited for comparisons in the setting of multicenter research and clinical follow-up studies. Moreover, based on an extensive population-based normative database embedded in the software, comparisons with age- and sex-adjusted controls are readily available, thus improving the validity of the analysis compared to previous literature, which mostly compared with small, if any, control cohorts.

The main findings of our study are that (i) ET and td Parkinson’s disease patients showed similar volumetric measurements in almost all segmented brain regions; (ii) comparisons with the normative database revealed marked atrophy in regions located mainly in the basal-ganglia-thalamocortical circuitry in both diseases and (iii) disease-related factors contributed to the volumes of occipital lobes in both diseases and to the volumes of temporal lobes in ET and the putamen in td Parkinson’s disease.

While ET is typically characterized by an action tremor of the upper extremity and the main features described in Parkinson’s disease are bradykinesia, rest tremor and rigidity, overlapping motor and non-motor symptoms have been described. Moreover, pathological and neuroimaging findings point to certain common pathophysiological features.

Historically, there have been few studies comparing volumetric measurements in ET and Parkinson’s disease patients, using different study designs and mostly manual MRI evaluation methods. Lin *et al*. compared volumetric differences in 10 ET, 10 Parkinson’s disease and 13 HC with VBM and found both decreased and increased volumes in brain areas involved in basal-ganglia-thalamocortical and cerebello-thalamocortical circuitries. In addition to atrophy, they also found increased volumes in the thalamus in Parkinson’s disease and in the middle temporal gyrus in ET, suggesting compensatory effects.^14^ In contrast, texture-based MR imaging analyses of the cerebellum of 280 Parkinson’s disease and 109 ET patients revealed significant differences in the inferior cerebellar cortex.^16^ However, both studies included Parkinson’s disease patients without distinguishing different subtypes. The use of different motor tests in ET and Parkinson’s disease further restricts the ability to link MRI alterations to motor phenotypes. Two previous studies compared td Parkinson’s disease and ET: in 45 ET patients, 45 td Parkinson’s disease patients and 45 controls voxel-based automated segmentation showed cerebellar atrophy in patients with ET and concomitant head tremor, but no differences were found between ET without head tremor, td Parkinson’s disease and HC.^17^ Comparing 15 patients with ET with resting tremor and td Parkinson’s disease using a MR support vector machine analysis with combined VBM and diffusion tensor imaging analysis, no single predictor was found to discriminate between the two groups in another study, but after combining all predictors (GM and WM volume, mean diffusivity and fractional anisotropy), the discrimination accuracy reached 100%.^15^ Consistent with these previous findings, we also found no significant differences in automatically measured brain (sub)volumes between our ET and td Parkinson’s disease patients. The only, but weak, significance was found in the frontal lobe contralateral to the less affected side. Nevertheless, subgroup analyses pointed to thalamic atrophy in ET_WR_ as well as mesencephal and putaminal atrophy in td Parkinson’s disease and ET_WR_. Furthermore, the effects were more pronounced in the volume contralateral to the more affected side. In both groups, the proportion of subjects with volume deviations from HC suggests the involvement of structures located mainly in the basal-ganglia-thalamocortical circuitry, which is linked to the basal ganglia, the supplementary motor area, the thalamus, the prefrontal cortex, the temporal cortex, hippocampus and occipital regions.^27^ In contrast, only few deviations within the cerebello-thalamocortical circuitry, including the cerebellum, premotor cortex, thalamus, prefrontal cortex and parietal cortex,^27^ could be found. Nevertheless, atrophy of the thalamus was significantly more pronounced in ET, which may be related to a greater involvement of the cerebello-thalamocortical circuitry. The findings within the basal-ganglia-thalamocortical circuitry indicate that ET and Parkinson’s disease may share several pathomechanism, which might explain overlapping symptoms, such as resting tremor.

In ET, the cerebellum in particular is thought to drive tremor activity. However, we did not detect significant differences compared with td Parkinson’s disease and HC, and in fact the corresponding volume was even reduced in the td Parkinson’s disease subcohort. Unfortunately, our segmentation algorithm covered the entire cerebellar volume and therefore may have generated averaging effects. Similarly, previous studies did not find differences in whole volume but in specific regions within the cerebellum such as the vermis or ET patients with head tremor only, suggesting a heterogeneous or more advanced pathological progress.^17,28^ Since few ET patients in our cohort presented with a concomitant head tremor, we did not perform a further subgroup analysis on this issue.

No difference was found between ET_P_ and ET_K_ in our subgroup analysis. This is consistent with a previous study demonstrating no regional differences in GM and WM volumes in 14 patients with postural tremor only and 13 patients with additional intention tremor. Nevertheless, the authors reported a relative expansion of GM in the region of the temporoparietal junction and the right middle occipital cortex compared with HC, and postulated a long-term result of adaptive reorganization compensating for the higher demands on visuospatial control of skilled movements in the setting of tremor.^8^ The software used in our study yields only negative deviations from the normative database, so comparisons along this line are infeasible.

To date, functional and structural neuroimaging studies investigating rest tremor in ET have yielded controversial results, including reduced connectivity in the cerebello-thalamocortical network^29^ or in various cortical areas and internal globus pallidus,^30^ decreased cerebellar GM diffusivity compared with ET without rest tremor,^31^ greater microstructural damage to the cerebellar WM,^32^ and decreased activation of the default mode network, putamen and cerebellum.^33^ Overall, these reports only suggest that abnormalities responsible for rest tremor might be localized in different brain regions than in action tremor. We also found no definite pattern when comparing td Parkinson’s disease, ET_R_ and ET_WR_, but the results of the different brain volumes and proportion of subjects with volume deviations from HC point to a stronger involvement of the thalamus in ET_WR_ compared with td Parkinson’s disease and ET_R_. The impact of the significantly reduced volumes in td Parkinson’s disease and ET_WR_ compared with ET_R_ remains, however, speculative.

Rest and kinetic tremor in ET are thought to occur with disease progression.^34,35^ The ET subgroups in this study did not differ in disease duration but both ET_R_ and ET_K_ had higher CRST scores indicating more severe tremor.

We further investigated whether disease-related factors, particularly duration of tremor (DOT) and tremor severity (measured by the CRST score), contributed to measured brain volumes. Moderate to strong effects could be demonstrated on the volumes of occipital lobes in both diseases and the volumes of temporal lobes in ET and the putamen in td Parkinson’s disease. Alterations in these regions were identified in previous studies, too^10,11,13^ and are considered to belong mainly to the basal-ganglia-thalamocortical network.^14^ Involvement of particular cortical regions could be a consequence of disease progression but also of non-motor features of the diseases.

Overall, demographics and clinical parameters were comparable in ET and td Parkinson’s disease as well as the subgroups. Nevertheless, the older age of Parkinson’s disease patients and shorter tremor duration might also contribute to the observed atrophy pattern. In addition, the severity of tremor may reflect more severe volumetric alterations. Considering that we included severely affected patients who sought MRgFUS treatment and performed careful tremor characterization using the same clinical score in ET and td Parkinson’s disease patients, we believe that our results are widely comparative. On the other hand, the results in our specific patient population with severe, medication-refractory tremor, do not allow general conclusions to be drawn for all ET and td Parkinson’s disease patients, especially those with good medication response.

This study also has some limitations. First, the diagnosis was mainly based on clinical criteria, but all patients had been previously diagnosed and usually provided additional records of diagnostic steps, e.g. DaTscan results, making misdiagnosis less likely. Second, the selection of td Parkinson’s disease patients was based on a self-defined tremor/non-tremor ratio. Nevertheless, this was carried out in a similar manner to previous studies using the UPDRS.^21^ Although methods using the MDS-UPDRS are available,^36^ we opted for differentiation from the akinetic-rigid phenotype instead of the postural instability/gait difficulty subtype mentioned therein. Third, we did not include our own control group. Therefore, metric comparisons of volumes and positive deviations indicative of compensatory hypertrophy, as mentioned above, were not possible. Nevertheless, we believe that comparisons with the extensive and age- and sex-matched normative database embedded in our software represent a valid control group. Fourth, inherent technical limitations may exist even with fully automated segmentation methods and depend on various confounding factors, such as slice thickness, MR imaging noise level, MR imaging orientation, field strength and anatomic boundary criteria, all of which may have an impact, albeit small, on the obtained measurements, even with a software product approved for clinical use.^9^ This is particularly relevant to studies performed at different imaging sites in the context of multicenter research. However, our study cohorts were initially enrolled prospectively and thus underwent standardized brain MRI, so that the influence of the usual aforementioned confounding factors can be largely excluded in our collective.

In conclusion, although the results do not allow precise differentiation of the various tremor types or of ET and Parkinson’s tremor on the basis of volumetric patterns, they indicate that neurodegenerative mechanisms including mainly the basal-ganglia-thalamocortical circuitry are involved in both diseases. In addition, disease-specific atrophy was found within the thalamus in ET_WR_ and in the mesencephalon and putamen in td Parkinson’s disease, suggesting a different degree of involvement of the circuitries in rest and action tremor. Especially in advanced tremor syndromes, invasive procedures such as DBS and MRgFUS, gain increasing relevance. Distinguishing different clinical phenotypes and their underlying pathology may be particularly crucial with regard to the different targets within the basal-ganglia-thalamocortical and cerebello-thalamocortical circuitry, such as the subthalamic nucleus or the ventral intermediate nucleus of the thalamus.

## Supplementary Material

fcad271_Supplementary_Data

## Data Availability

The data that support the findings of this study are available from the corresponding author, upon reasonable request.
