# Supplementary material for: Artificial-intelligence-based MRI brain volumetry in patients with essential tremor and tremor-dominant Parkinson’s disease
Source: Brain Commun. 2023 Oct 13;5(6):fcad271. doi: 10.1093/braincomms/fcad271 (PMC10631860; doi:10.1093/braincomms/fcad271)
Supplement: fcad271_Supplementary_Data [file fcad271_supplementary_data.docx]

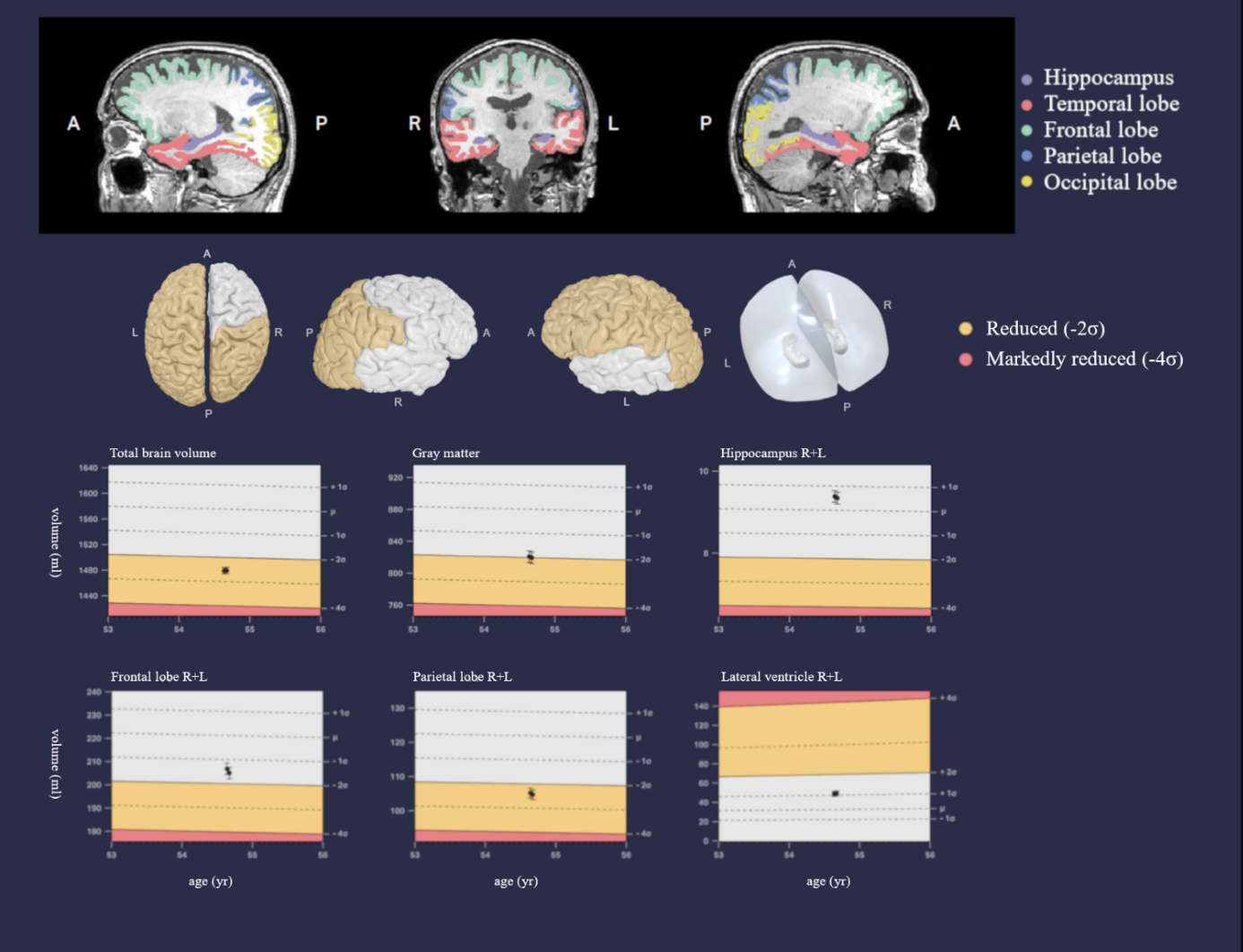


**Supplementary Figure 1:** Example of a fully-automated VBM by the AI-powered software mdbrain v1.1.1. Besides of volumes of several cortical and subcortical areas, the software provides deviations of all volumes compared to a validated norm collective (reported as deviation of 2 or 4 standard deviations).

**Supplementary Table 1 Percentage proportion of ET and PD patients with volume deviations compared to the norm collective**

|  | **ET (n=61)** | **PD (n=29)** | **p-value^#^** |
| --- | --- | --- | --- |
|  | **Mean** | **Mean** |  |
| **Total brain volume** | 2 | 0 | 0.49 |
| **White matter** | 3 | 0 | 0.32 |
| **Gray matter** | 2 | 0 | 0.49 |
| **Cortical gray matter** | 2 | 0 | 0.49 |
| **Frontal lobe ^a^** |  |  |  |
| More affected | 0 | 0 | n.a. |
| Less affected | 0 | 0 | n.a. |
| **Parietal lobe ^a^** |  |  |  |
| More affected | 3 | 0 | 0.32 |
| Less affected | 2 | 0 | 0.49 |
| **Precuneus lobe ^a^** |  |  |  |
| More affected | 0 | 0 | n.a. |
| Less affected | 3 | 3 | 0.97 |
| **Occipital lobe ^a^** |  |  |  |
| More affected | 7 | 3 | 0.55 |
| Less affected | 5 | 7 | 0.70 |
| **Temporal lobe ^a^** |  |  |  |
| More affected | 0 | 0 | n.a. |
| Less affected | 0 | 0 | n.a. |
| **Hippocampus ^a^** |  |  |  |
| More affected | 8 | 0 | 0.11 |
| Less affected | 0 | 3 | 0.15 |
| **Parahippocampus ^a^** |  |  |  |
| More affected | 0 | 0 | n.a. |
| Less affected | 0 | 0 | n.a. |
| **Entorhinal lobe ^a^** |  |  |  |
| More affected | 5 | 0 | 0.22 |
| Less affected | 2 | 0 | 0.49 |
| **Caudate ^a^** |  |  |  |
| More affected | 13 | 0 | 0.04* |
| Less affected | 5 | 0 | 0.22 |
| **Putamen ^a^** |  |  |  |
| More affected | 12 | 10 | 0.87 |
| Less affected | 7 | 14 | 0.26 |
| **Pallidum ^a^** |  |  |  |
| More affected | 20 | 17 | 0.78 |
| Less affected | 20 | 10 | 0.27 |
| **Thalamus ^a^** |  |  |  |
| More affected | 10 | 0 | 0.08 |
| Less affected | 16 | 3 | 0.08 |
| **Brainstem** | 3 | 3 | 0.97 |
| **Mesencephalon** | 7 | 10 | 0.53 |
| **Pons** | 2 | 3 | 0.59 |
| **Cerebellum** | 0 | 0 | n.a. |
| **Lateral ventricle ^a^** |  |  |  |
| More affected | 13 | 3 | 0.21 |
| Less affected | 5 | 3 | 0.55 |
| **Third ventricle** | 26 | 7 | 0.03* |
| **Fourth ventricle** | 8 | 0 | 0.11 |
| Percentage proportion of ET and PD patients with volume deviations compared to the norm collective.  # p-value of the χ 2.  * p-value <. 0.05 was considered statistically significant.  a the more/less affected brain volumes is defined as the volume contralateral to the more/less affected tremor side.  Abbreviations: ET= essential tremor. PD=Parkinson’s Disease. | | | |

**Supplementary Table 2 Results of volumetric MRI analysis in ET and PD patients**

|  | ET (n=61) | | PD (n=29) | |  | p-value^#^ |
| --- | --- | --- | --- | --- | --- | --- |
|  | Mean | SD | Mean | SD | % Δ |  |
| ICV | 1.000 |  | 1.000 |  |  |  |
| Total brain volume | 0.963 | 0.0188 | 0.972 | 0.0139 | 1 | 0.534 |
| White matter | 0.422 | 0.0170 | 0.431 | 0.0197 | 2 | 0.102 |
| Gray matter | 0.541 | 0.0178 | 0.541 | 0.0174 | 0 | 0.180 |
| Cortical gray matter | 0.369 | 0.0146 | 0.373 | 0.0155 | 1 | 0.320 |
| Frontal lobe ^a^  Total | 0.133 | 0.0075 | 0.136 | 0.0072 | 2 | 0.071 |
| More affected | 0.066 | 0.0039 | 0.068 | 0.0033 | 2 | 0.161 |
| Less affected | 0.067 | 0.0038 | 0.069 | 0.0042 | 2 | 0.049* |
| Parietal lobe ^a^  Total | 0.073 | 0.0046 | 0.074 | 0.0044 | 1 | 0.634 |
| More affected | 0.037 | 0.0024 | 0.037 | 0.0023 | 1 | 0.490 |
| Less affected | 0.036 | 0.0028 | 0.037 | 0.0024 | 1 | 0.871 |
| Precuneus lobe ^a^  Total | 0.018 | 0.0013 | 0.018 | 0.0014 | 1 | 0.331 |
| More affected | 0.009 | 0.0007 | 0.009 | 0.0007 | 1 | 0.386 |
| Less affected | 0.009 | 0.0007 | 0.009 | 0.0008 | 2 | 0.387 |
| Occipital lobe ^a^  Total | 0.053 | 0.0033 | 0.055 | 0.0034 | 3 | 0.078 |
| More affected | 0.027 | 0.0021 | 0.028 | 0.0021 | 2 | 0.889 |
| Less affected | 0.026 | 0.0023 | 0.027 | 0.0025 | 3 | 0.480 |
| Temporal lobe ^a^  Total | 0.109 | 0.0055 | 0.109 | 0.0052 | -1 | 0.765 |
| More affected | 0.054 | 0.0033 | 0.054 | 0.0033 | 0 | 0.882 |
| Less affected | 0.055 | 0.0033 | 0.055 | 0.0027 | -1 | 0.713 |
| Hippocampus ^a^  Total | 0.006 | 0.0005 | 0.006 | 0.0005 | 1 | 0.696 |
| More affected | 0.003 | 0.0003 | 0.003 | 0.0003 | 1 | 0.722 |
| Less affected | 0.003 | 0.0003 | 0.003 | 0.0003 | 1 | 0.804 |
| Parahippocampus ^a^  Total | 0.005 | 0.0004 | 0.005 | 0.0005 | -1 | 0.834 |
| More affected | 0.003 | 0.0002 | 0.003 | 0.0003 | 0 | 0.966 |
| Less affected | 0.003 | 0.0002 | 0.003 | 0.0002 | -1 | 0.703 |
| Entorhinal lobe ^a^  Total | 0.004 | 0.0004 | 0.004 | 0.0003 | 1 | 0.193 |
| More affected | 0.002 | 0.0003 | 0.002 | 0.0002 | 2 | 0.136 |
| Less affected | 0.002 | 0.0002 | 0.002 | 0.0002 | 0 | 0.449 |
| Caudate ^a^  Total | 0.005 | 0.0009 | 0.005 | 0.0007 | -3 | 0.872 |
| More affected | 0.003 | 0.0005 | 0.002 | 0.0003 | -5 | 0.464 |
| Less affected | 0.003 | 0.0005 | 0.003 | 0.0005 | -2 | 0.687 |
| Putamen ^a^  Total | 0.007 | 0.0007 | 0.006 | 0.0005 | -4 | 0.309 |
| More affected | 0.003 | 0.0004 | 0.003 | 0.0003 | -3 | 0.241 |
| Less affected | 0.003 | 0.0004 | 0.003 | 0.0003 | -5 | 0.428 |
| Pallidum ^a^  Total | 0.002 | 0.0002 | 0.002 | 0.0002 | 1 | 0.133 |
| More affected | 0.001 | 0.0001 | 0.001 | 0.0001 | 1 | 0.144 |
| Less affected | 0.001 | 0.0001 | 0.001 | 0.0001 | 2 | 0.164 |
| Thalamus ^a^  Total | 0.012 | 0.0008 | 0.013 | 0.0007 | 2 | 0.857 |
| More affected | 0.006 | 0.0005 | 0.006 | 0.0004 | 3 | 0.830 |
| Less affected | 0.006 | 0.0005 | 0.006 | 0.0004 | 2 | 0.602 |
| Brainstem | 0.022 | 0.0016 | 0.022 | 0.0015 | -1 | 0.396 |
| Mesencephalon | 0.006 | 0.0004 | 0.006 | 0.0004 | -2 | 0.287 |
| Pons | 0.012 | 0.0010 | 0.012 | 0.0010 | 0 | 0.639 |
| Cerebellum | 0.085 | 0.0067 | 0.083 | 0.0048 | -2 | 0.565 |
| Ventricle volume | 0.037 | 0.0188 | 0.028 | 0.0139 | -26 | 0.534 |
| Lateral ventricle ^a^  Total | 0.035 | 0.0183 | 0.026 | 0.0136 | -27 | 0.554 |
| More affected | 0.018 | 0.0095 | 0.013 | 0.0076 | -28 | 0.307 |
| Less affected | 0.017 | 0.0091 | 0.012 | 0.0064 | -26 | 0.919 |
| Third ventricle | 0.001 | 0.0005 | 0.001 | 0.0014 | -9 | 0.703 |
| Fourth ventricle | 0.001 | 0.0003 | 0.001 | 0.0003 | -19 | 0.492 |
| Relative volumes of the different brain volumes for ET and PD patients. All volumes have been normalized to the intracranial volume (ICV).  # p-value of the ANCOVA performed with age, sex, age of onset and disease duration as covariates.  * p-value <0.005 was considered statistically significant.  ^a^ the more/less affected brain volumes is defined as the volume contralateral to the more/less affected tremor side.  Abbreviations: ET=Essential Tremor. PD=Parkinson’s Disease. SD=Standard deviation. % Δ=percentage difference of the mean of PD patients to the mean of ET patients. ICV=intracranial volume | | | | | | |

**Supplementary Table 3 Results of volumetric MRI analysis in ET with and without rest tremor**

|  | **I. ET_R_ (n=29)** | | **II. ET_WR_ (n=32)** | | **III. PD (n=29)** | | **p-value^#^** | **Post-hoc**^†^ | | |
| --- | --- | --- | --- | --- | --- | --- | --- | --- | --- | --- |
|  | **Mean** | **SD** | **Mean** |  | **Mean** | **SD** |  | **I vs. II** | **I vs. III** | **II vs. III** |
| **ICV** | 1.000 |  | 1.000 |  | 1.000 |  |  |  |  |  |
| **Total brain volume** | 0,964 | 0,0185 | 0,961 | 0,0193 | 0,972 | 0,0139 | 0.019* | 0.807 | 0.049* | 0.002** |
| **White matter** | 0,419 | 0,0165 | 0,425 | 0,0171 | 0,431 | 0,0197 | 0.545 | 0.464 | 0.033 | 0.592 |
| **Gray matter** | 0,546 | 0,0189 | 0,536 | 0,0155 | 0,541 | 0,0174 | 0.130 | 0.042 | 0.820 | 0.454 |
| **Cortical gray matter** | 0,371 | 0,0167 | 0,367 | 0,0123 | 0,373 | 0,0155 | 0.137 | 0.488 | 1.000 | 0.113 |
| **Frontal lobe ^a^**  Total | 0,134 | 0,0079 | 0,133 | 0,0072 | 0,136 | 0,0072 | 0.166 | 1.000 | 0.507 | 0.095 |
| More affected | 0,067 | 0,0042 | 0,066 | 0,0037 | 0,068 | 0,0033 | 0.119 | 1.000 | 0.568 | 0.123 |
| Less affected | 0,067 | 0,0040 | 0,067 | 0,0037 | 0,069 | 0,0041 | 0.154 | 1.000 | 0.557 | 0.111 |
| **Parietal lobe ^a^**  Total | 0,074 | 0,0051 | 0,073 | 0,0041 | 0,074 | 0,0044 | 0.349 | 0.413 | 1.000 | 0.447 |
| More affected | 0,037 | 0,0022 | 0,036 | 0,0025 | 0,037 | 0,0023 | 0.171 | 0.453 | 1.000 | 0.337 |
| Less affected | 0,037 | 0,0033 | 0,036 | 0,0022 | 0,037 | 0,0024 | 0.692 | 0.661 | 1.000 | 1.000 |
| **Precuneus lobe ^a^**  Total | 0,017 | 0,0014 | 0,018 | 0,0011 | 0,018 | 0,0014 | 0.323 | 1.000 | 0.759 | 1.000 |
| More affected | 0,009 | 0,0008 | 0,009 | 0,0007 | 0,009 | 0,0007 | 0.616 | 1.000 | 1.000 | 1.000 |
| Less affected | 0,009 | 0,0008 | 0,009 | 0,0006 | 0,009 | 0,0008 | 0.210 | 1.000 | 0.473 | 1.000 |
| **Occipital lobe ^a^**  Total | 0,053 | 0,0039 | 0,053 | 0,0027 | 0,055 | 0,0034 | 0.638 | 1.000 | 0.081 | 0.423 |
| More affected | 0,027 | 0,0022 | 0,027 | 0,0020 | 0,028 | 0,0021 | 0.902 | 1.000 | 0.324 | 1.000 |
| Less affected | 0,026 | 0,0025 | 0,026 | 0,0022 | 0,027 | 0,0025 | 0.616 | 1.000 | 0.501 | 0.271 |
| **Temporal lobe ^a^**  Total | 0,110 | 0,0067 | 0,108 | 0,0040 | 0,109 | 0,0052 | 0.330 | 0.322 | 0.606 | 1.000 |
| More affected | 0,055 | 0,0041 | 0,054 | 0,0023 | 0,054 | 0,0033 | 0.584 | 0.461 | 0.904 | 1.000 |
| Less affected | 0,056 | 0,0034 | 0,055 | 0,0031 | 0,055 | 0,0027 | 0.260 | 0.430 | 0.628 | 1.000 |
| **Hippocampus ^a^**  Total | 0,006 | 0,0006 | 0,006 | 0,0004 | 0,006 | 0,0005 | 0.004** | 0.044* | 1.000 | 0.261 |
| More affected | 0,003 | 0,0004 | 0,003 | 0,0003 | 0,003 | 0,0003 | 0.045* | 0.163 | 1.000 | 0.564 |
| Less affected | 0,003 | 0,0003 | 0,003 | 0,0003 | 0,003 | 0,0003 | 0.017* | 0.161 | 1.000 | 0.526 |
| **Parahippocampus ^a^**  Total | 0,005 | 0,0004 | 0,005 | 0,0004 | 0,005 | 0,0005 | 0.312 | 1.000 | 1.000 | 1.000 |
| More affected | 0,003 | 0,0002 | 0,003 | 0,0002 | 0,003 | 0,0003 | 0.705 | 1.000 | 1.000 | 1.000 |
| Less affected | 0,003 | 0,0002 | 0,003 | 0,0002 | 0,003 | 0,0002 | 0.161 | 1.000 | 0.895 | 1.000 |
| **Entorhinal lobe ^a^**  Total | 0,004 | 0,0005 | 0,004 | 0,0004 | 0,004 | 0,0003 | 0.882 | 1.000 | 1.000 | 0.903 |
| More affected | 0,002 | 0,0003 | 0,002 | 0,0002 | 0,002 | 0,0002 | 0.789 | 1.000 | 1.000 | 1.000 |
| Less affected | 0,002 | 0,0002 | 0,002 | 0,0002 | 0,002 | 0,0002 | 0.461 | 0.284 | 1.000 | 1.000 |
| **Caudate ^a^**  Total | 0,005 | 0,0009 | 0,005 | 0,0010 | 0,005 | 0,0007 | 0.974 | 1.000 | 0.838 | 1.000 |
| More affected | 0,002 | 0,0004 | 0,002 | 0,0006 | 0,002 | 0,0003 | 0.746 | 1.000 | 0.448 | 1.000 |
| Less affected | 0,003 | 0,0005 | 0,003 | 0,0005 | 0,003 | 0,0004 | 0.927 | 1.000 | 1.000 | 1.000 |
| **Putamen ^a^**  Total | 0,007 | 0,0006 | 0,006 | 0,0008 | 0,006 | 0,0005 | 0.101 | 0.065 | 0.015 | 1.000 |
| More affected | 0,003 | 0,0003 | 0,003 | 0,0004 | 0,003 | 0,0003 | 0.038* | 0.044* | 0.031* | 1.000 |
| Less affected | 0,003 | 0,0003 | 0,003 | 0,0004 | 0,003 | 0,0003 | 0.218 | 0.126 | 0.011 | 0.842 |
| **Pallidum ^a^**  Total | 0,002 | 0,0002 | 0,002 | 0,0002 | 0,002 | 0,0002 | 0.238 | 0.658 | 1.000 | 0.676 |
| More affected | 0,001 | 0,0001 | 0,001 | 0,0001 | 0,001 | 0,0001 | 0.293 | 0.741 | 1.000 | 0.882 |
| Less affected | 0,001 | 0,0001 | 0,001 | 0,0001 | 0,001 | 0,0001 | 0.249 | 0.709 | 1.000 | 0.622 |
| **Thalamus ^a^**  Total | 0,012 | 0,0008 | 0,012 | 0,0008 | 0,013 | 0,0007 | 0.016* | 0.408 | 1.000 | 0.092 |
| More affected | 0,006 | 0,0005 | 0,006 | 0,0004 | 0,006 | 0,0004 | 0.003** | 0.177 | 1.000 | 0.018* |
| Less affected | 0,006 | 0,0004 | 0,006 | 0,0005 | 0,006 | 0,0004 | 0.105 | 0.963 | 1.000 | 0.466 |
| **Brainstem** | 0,022 | 0,0015 | 0,022 | 0,0016 | 0,022 | 0,0015 | 0.181 | 0.141 | 0.498 | 1.000 |
| **Mesencephalon** | 0,006 | 0,0004 | 0,006 | 0,0004 | 0,006 | 0,0004 | 0.014* | 0.018* | 0.041* | 1.000 |
| **Pons** | 0,012 | 0,0010 | 0,012 | 0,0010 | 0,012 | 0,0010 | 0.387 | 0.377 | 1.000 | 1.000 |
| **Cerebellum** | 0,087 | 0,0071 | 0,083 | 0,0058 | 0,082 | 0,0048 | 0.230 | 0.028* | 0.028* | 1.000 |
| **Ventricle volume** | 0,036 | 0,0185 | 0,039 | 0,0193 | 0,028 | 0,0139 | 0.019* | 0.807 | 0.049* | 0.002** |
| **Lateral ventricle ^a^**  Total | 0,033 | 0,0180 | 0,037 | 0,0188 | 0,026 | 0,0136 | 0.019* | 0.812 | 0.054 | 0.003** |
| More affected | 0,018 | 0,0099 | 0,019 | 0,0091 | 0,013 | 0,0076 | 0.040* | 1.000 | 0.045* | 0.004** |
| Less affected | 0,016 | 0,0082 | 0,017 | 0,0099 | 0,012 | 0,0064 | 0.011* | 0.633 | 0.086 | 0.003** |
| **Third ventricle** | 0,001 | 0,0005 | 0,001 | 0,0005 | 0,001 | 0,0014 | 0.820 | 1.000 | 1.000 | 1.000 |
| **Fourth ventricle** | 0,001 | 0,0003 | 0,001 | 0,0004 | 0,001 | 0,0003 | 0.126 | 1.000 | 0.079 | 0.024 |
| Relative volumes of the different brain volumes for ET patients with and without rest tremor. All volumes have been normalized to the intracranial volume (ICV). Percentage volume differences of PD to ET patients were colored in shades of red (volume loss), white (control level) and shades of blue (volume gain).  # p-value of the ANCOVA performed with age, sex, age of onset and disease duration as covariates.  ^†^ post-hoc analysis was performed with the Bonferroni test.  * p-value < 0.005 was considered statistically significant.  ^a^ the more/less affected brain volumes is defined as the volume contralateral to the more/less affected tremor side.  Abbreviations: ET_R_ = essential tremor with rest tremor. ET_WR_= essential tremor without rest tremor. SD=Standard deviation. % Δ=percentage difference of the mean of ET_WR_ patients to the mean of ET_R_ patients. ICV=intracranial volume | | | | | | | | | | |

**Supplementary Table 4 Results of volumetric MRI analysis in ET with more pronounced postural or kinetic tremor**

|  | **ET_P_ (n=20)** | | **ET_K_ (n=25)** | | **p-value^#^** |
| --- | --- | --- | --- | --- | --- |
|  | **Mean** | **SD** | **Mean** | **SD** |  |
| **ICV** | 1.000 |  | 1.000 |  |  |
| **Total brain volume** | 0.966 | 0.0168 | 0.968 | 0.0145 | 0.447 |
| **White matter** | 0.422 | 0.0167 | 0.427 | 0.0170 | 0.853 |
| **Gray matter** | 0.544 | 0.0162 | 0.541 | 0.0188 | 0.410 |
| **Cortical gray matter** | 0.370 | 0.0125 | 0.370 | 0.0141 | 0.279 |
| **Frontal lobe ^a^**  Total | 0.135 | 0.0066 | 0.133 | 0.0068 | 0.471 |
| More affected | 0.067 | 0.0036 | 0.066 | 0.0036 | 0.397 |
| Less affected | 0.068 | 0.0034 | 0.067 | 0.0037 | 0.663 |
| **Parietal lobe ^a^**  Total | 0.074 | 0.0045 | 0.073 | 0.0051 | 0.242 |
| More affected | 0.037 | 0.0021 | 0.037 | 0.0027 | 0.471 |
| Less affected | 0.037 | 0.0027 | 0.037 | 0.0029 | 0.200 |
| **Precuneus lobe ^a^**  Total | 0.018 | 0.0011 | 0.018 | 0.0013 | 0.065 |
| More affected | 0.009 | 0.0006 | 0.009 | 0.0008 | 0.117 |
| Less affected | 0.009 | 0.0007 | 0.009 | 0.0007 | 0.117 |
| **Occipital lobe ^a^**  Total | 0.053 | 0.0023 | 0.054 | 0.0038 | 0.963 |
| More affected | 0.027 | 0.0017 | 0.027 | 0.0025 | 0.591 |
| Less affected | 0.026 | 0.0021 | 0.027 | 0.0025 | 0.655 |
| **Temporal lobe ^a^**  Total | 0.109 | 0.0042 | 0.110 | 0.0053 | 0.379 |
| More affected | 0.054 | 0.0030 | 0.055 | 0.0033 | 0.424 |
| Less affected | 0.055 | 0.0025 | 0.055 | 0.0033 | 0.606 |
| **Hippocampus ^a^**  Total | 0.006 | 0.0005 | 0.006 | 0.0005 | 0.616 |
| More affected | 0.003 | 0.0003 | 0.003 | 0.0003 | 0.581 |
| Less affected | 0.003 | 0.0003 | 0.003 | 0.0003 | 0.834 |
| **Parahippocampus ^a^**  Total | 0.005 | 0.0005 | 0.005 | 0.0003 | 0.710 |
| More affected | 0.003 | 0.0003 | 0.003 | 0.0002 | 0.908 |
| Less affected | 0.003 | 0.0002 | 0.003 | 0.0002 | 0.363 |
| **Entorhinal lobe ^a^**  Total | 0.004 | 0.0005 | 0.004 | 0.0004 | 0.271 |
| More affected | 0.002 | 0.0003 | 0.002 | 0.0002 | 0.830 |
| Less affected | 0.002 | 0.0002 | 0.002 | 0.0002 | 0.051 |
| **Caudate ^a^**  Total | 0.005 | 0.0009 | 0.005 | 0.0008 | 0.690 |
| More affected | 0.003 | 0.0005 | 0.002 | 0.0004 | 0.564 |
| Less affected | 0.003 | 0.0005 | 0.002 | 0.0004 | 0.899 |
| **Putamen ^a^**  Total | 0.007 | 0.0006 | 0.007 | 0.0006 | 0.453 |
| More affected | 0.004 | 0.0003 | 0.003 | 0.0004 | 0.724 |
| Less affected | 0.004 | 0.0004 | 0.003 | 0.0003 | 0.273 |
| **Pallidum ^a^**  Total | 0.002 | 0.0002 | 0.002 | 0.0002 | 0.242 |
| More affected | 0.001 | 0.0001 | 0.001 | 0.0001 | 0.381 |
| Less affected | 0.001 | 0.0001 | 0.001 | 0.0001 | 0.179 |
| **Thalamus ^a^**  Total | 0.013 | 0.0007 | 0.013 | 0.0008 | 0.821 |
| More affected | 0.006 | 0.0004 | 0.006 | 0.0005 | 0.692 |
| Less affected | 0.006 | 0.0004 | 0.006 | 0.0004 | 1.000 |
| **Brainstem** | 0.022 | 0.0014 | 0.022 | 0.0016 | 0.883 |
| **Mesencephalon** | 0.006 | 0.0004 | 0.006 | 0.0004 | 0.978 |
| **Pons** | 0.012 | 0.0010 | 0.012 | 0.0011 | 0.889 |
| **Cerebellum** | 0.085 | 0.0056 | 0.085 | 0.0063 | 0.969 |
| **Ventricle volume** | 0.034 | 0.0168 | 0.032 | 0.0145 | 0.447 |
| **Lateral ventricle ^a^**  Total | 0.032 | 0.0162 | 0.029 | 0.0140 | 0.455 |
| More affected | 0.017 | 0.0087 | 0.016 | 0.0069 | 0.478 |
| Less affected | 0.015 | 0.0077 | 0.014 | 0.0073 | 0.454 |
| **Third ventricle** | 0.001 | 0.0005 | 0.001 | 0.0005 | 0.319 |
| **Fourth ventricle** | 0.001 | 0.0003 | 0.001 | 0.0004 | 0.788 |
| Relative volumes of the different brain volumes for ET patients with more pronounced postural or kinetic tremor. All volumes have been normalized to the intracranial volume (ICV). Percentage volume differences of PD to ET patients were colored in shades of red (volume loss), white (control level) and shades of blue (volume gain).  # p-value of the ANCOVA performed with age, sex, age of onset and disease duration as covariates.  * p-value <. 0.005 was considered statistically significant.  a the more/less affected brain volumes is defined as the volume contralateral to the more/less affected tremor side.  Abbreviations: ET_P_= essential tremor with pronounced postural tremor. ET_K_= essential tremor with pronounced kinetic tremor. SD=Standard deviation. % Δ=percentage difference of the mean of ET_K_ patients to the mean of ET_P_ patients. ICV=intracranial volume | | | | | |

**Supplementary Table 5 Disease-related correlates of volumetric measurements in ET and PD**

|  | **ET (n=61)** | | | | **PD (n=29)** | | | |
| --- | --- | --- | --- | --- | --- | --- | --- | --- |
|  | **Block 1** | | **Block 2** | | **Block 1** | | **Block 2** | |
|  | **Age** | **Sex** | **DOT** | **CTRS** | **Age** | **Sex** | **DOT** | **CTRS** |
| **Total brain volume**  Std. β ^a^  R²  F  ΔR²  ΔF | -.65**  0.43  22.07** | -.31** | .24*  0.51  0.09  5.25** | .21* | -.69**  0.43  11.26** | -.26 | -.29  0.48  0.08  2.14 | -.01 |
| **White matter**  Std. β ^a^  R²  F  ΔR²  ΔF | -.10  -0.02  0.42 | .05 | .19  -0.01  0.04  1.20 | -.09 | -.39  0.08  2.10 | -.07 | .11  0.07  0.06  0.86 | -.25 |
| **Gray matter**  Std. β ^a^  R²  F  ΔR²  ΔF | -.56**  0.34  15.29** | -.35** | .06  0.40  0.08  3.60* | .29* | -.12  -0.06  0.22 | -.13 | -.36  0.02  0.15  2.08 | .28 |
| **Cortical gray matter**  Std. β ^a^  R²  F  ΔR²  ΔF | -.66**  0.45  23.64** | -.31** | .08  0.47  0.04  2.06 | .20 | -.25  -0.01  0.82 | -.14 | -.42  0.09  0.16  2.44 | .21 |
| **Frontal lobe**  Std. β ^a^  R²  F  ΔR²  ΔF | -.67**  0.54  33.63** | -.36** | .07  0.53  0.01  0.52 | .07 | -.13  -0.03  0.56 | .08 | -.35  0.11  0.20  2.96 | .40 |
| **Parietal lobe**  Std. β ^a^  R²  F  ΔR²  ΔF | -.56**  0.34  15.10** | -.24* | -.09  0.32  0.01  0.36 | .03 | -.38  0.07  1.97 | -.15 | -.35  0.11  0.11  1.64 | .09 |
| **Precuneus lobe**  Std. β ^a^  R²  F  ΔR²  ΔF | -.36  0.07  3.09 | -.04 | .13  0.06  0.03  0.85 | .12 | -.03  -0.08  0.01 | -.07 | -.36  -0.03  0.12  1.58 | .02 |
| **Occipital lobe**  Std. β ^a^  R²  F  ΔR²  ΔF | -.06  0.02  1.67 | -.22 | .36**  0.13  0.13  4.22* | .08 | -.06  -0.01  0.83 | -.34 | -.50*  0.19  0.24  4.03* | -.02 |
| **Temporal lobe**  Std. β ^a^  R²  F  ΔR²  ΔF | -.37  0.03  1.89 | .01 | -.04  0.14  0.13  4.37* | .38** | -.22  -0.01  0.86 | -.20 | -.19  -0.06  0.04  0.44 | < -.01 |
| **Hippocampus**  Std. β ^a^  R²  F  ΔR²  ΔF | -.42**  0.08  3.46* | -.05 | .12  0.14  0.09  2.94 | .29* | -.26  -0.01  0.88 | -.14 | -.28  <0.01  0.09  1.16 | -.05 |
| **Parahippocampus**  Std. β ^a^  R²  F  ΔR²  ΔF | .07  -0.02  0.41 | .07 | .06  -0.05  0.02  0.40 | .12 | .09  -0.07  0.13 | .10 | .19  -0.12  0.04  0.46 | .03 |
| **Entorhinal lobe**  Std. β ^a^  R²  F  ΔR²  ΔF | -.02  -0.03  0.11 | .08 | .30  0.03  0.09  2.71 | .07 | -.48  0.14  3.21 | -.16 | -.15  0.10  0.03  0.48 | -.07 |
| **Caudate**  Std. β ^a^  R²  F  ΔR²  ΔF | .21  0.08  3.58* | -.33* | -.16  0.11  0.06  1.87 | -.20 | .14  -0.04  0.43 | .16 | .12  -0.12  0.01  0.15 | -.01 |
| **Putamen**  Std. β ^a^  R²  F  ΔR²  ΔF | -.11  0.02  1.54 | -.20 | -.06  -0.01  0.01  0.14 | -.04 | .09  -0.07  0.09 | .09 | .18  0.27  0.37  6.84** | .53** |
| **Pallidum**  Std. β ^a^  R²  F  ΔR²  ΔF | -.03  -0.01  0.83 | -.16 | .17  -0.01  0.03  0.81 | .06 | -.41*  0.14  3.15* | .14 | .10  0.10  0.03  0.45 | .11 |
| **Thalamus**  Std. β ^a^  R²  F  ΔR²  ΔF | -.30*  0.10  4.22* | -.25 | .13  0.10  0.03  0.99 | .14 | -.25  0.02  1.29 | -.24 | -.09  -0.05  0.01  0.16 | -.06 |
| **Brainstem**  Std. β ^a^  R²  F  ΔR²  ΔF | .36*  0.10  3.96* | .07 | .06  0.07  <0.01  0.13 | -.02 | .11  -0.04  0.49 | .18 | .03  -0.08  0.04  0.51 | .20 |
| **Mesencephalon**  Std. β ^a^  R²  F  ΔR²  ΔF | .30  0.06  2.63 | .10 | .01  0.02  0.01  0.16 | -.08 | -.03  0.03  1.44 | .34 | .15  -0.03  0.02  0.30 | .01 |
| **Pons**  Std. β ^a^  R²  F  ΔR²  ΔF | .35*  0.09  4.04* | .07 | .08  0.07  0.01  0.23 | -.01 | .19  -0.04  0.52 | .11 | .03  -0.07  0.05  0.64 | .21 |
| **Cerebellum**  Std. β ^a^  R²  F  ΔR²  ΔF | -.12  0.01  1.19 | -.18 | .01  0.03  0.05  1.34 | .23 | -.17  -0.04  0.53 | -.14 | -.02  -0.05  0.07  0.84 | .26 |
| **Lateral ventricle**  Std. β ^a^  R²  F  ΔR²  ΔF | .65**  0.43  21.71** | .31** | -.24*  0.50  0.09  5.00* | -.20* | .68**  0.41  10.45** | .25 | .28  0.45  0.08  1.94 | .02 |
| **Third ventricle**  Std. β ^a^  R²  F  ΔR²  ΔF | .67**  0.43  22.35** | .32** | -.15  0.51  0.09  5.12** | -.28** | .23  -0.01  0.81 | .15 | .12  -0.08  0.02  0.20 | -.09 |
| **Fourth ventricle**  Std. β ^a^  R²  F  ΔR²  ΔF | .26  <0.01  1.12 | -.04 | -.25  0.06  0.09  2.61 | -.19 | .25  -0.01  0.92 | .17 | .24  -0.03  0.05  0.71 | -.13 |
| Hierarchical multiple regression analyses for ET and PD patients. Regression analyses consisted of two steps: 1) demographic variables (age and sex) were entered as the first block, and 2) disease-related factors (duration of tremor and tremor severity measured by the CTRS) were entered in the second block.  ** p<0.01, * p<0.05  ^a^ all standarized regression coefficients are from the final step in the analyses.  Abbreviations: ET=Essential Tremor, PD=Parkinson’s Disease, DOT = Duration of tremor, CRST = Clinical Rating Scale for Tremor | | | | | | | | |

**Supplementary Table 6 Disease-related correlates of volumetric measurements in ET patients**

| **ET (n=61)** | | | | | | | | |
| --- | --- | --- | --- | --- | --- | --- | --- | --- |
|  | **Contralateral to more affected side** | | | | **Contralateral to less affected side** | | | |
|  | **Block 1** | | **Block 2** | | **Block 1** | | **Block 2** | |
|  | **Age** | **Sex** | **DOT** | **CTRS** | **Age** | **Sex** | **DOT** | **CTRS** |
| **Frontal lobe**  Std. β ^a^  R²  F  ΔR²  ΔF | -.60**  0.46  25.24** | -.36** | .04  0.45  < 0.01  0.21 | .05 | -.68**  0.53  33.08** | -.34** | .08  0.53  0.01  0.78 | .07 |
| **Parietal lobe**  Std. β ^a^  R²  F  ΔR²  ΔF | -.50**  0.25  10.40** | -.18 | -.12  0.24  0.02  0.63 | .04 | -.48**  0.27  11.26** | -.24* | -.04  0.24  < 0.01  0.06 | -.02 |
| **Precuneus lobe**  Std. β ^a^  R²  F  ΔR²  ΔF | -.26  0.03  1.86 | -.06 | .09  < 0.01  0.01  0.30 | .05 | -.34  0.07  2.95 | -.02 | .12  0.05  0.02  0.69 | .09 |
| **Occipital lobe**  Std. β ^a^  R²  F  ΔR²  ΔF | -.19  0.03  1.74 | -.18 | .27  0.10  0.10  3.17 | .18 | .08  < -0.01  0.92 | -.15 | .26  0.03  0.07  1.97 | .02 |
| **Temporal lobe**  Std. β ^a^  R²  F  ΔR²  ΔF | -.18  -0.03  0.27 | .04 | -.01  0.05  0.10  3.01 | .33 | -.39  0.07  3.04 | -.05 | -.12  0.12  0.09  2.71 | .29 |
| **Hippocampus**  Std. β ^a^  R²  F  ΔR²  ΔF | -.33  0.05  2.40 | -.07 | .09  0.08  0.07  2.04 | .25 | -.35  0.03  1.99 | -.03 | .04  0.09  0.09  2.81 | .31 |
| **Parahippocampus**  Std. β ^a^  R²  F  ΔR²  ΔF | .07  -0.02  0.35 | -.02 | .02  -0.04  0.02  0.53 | .14 | .04  -0.01  0.34 | .15 | .06  -0.02  0.03  0.73 | .15 |
| **Entorhinal lobe**  Std. β ^a^  R²  F  ΔR²  ΔF | .08  -0.03  0.24 | < .01 | .25  < -0.01  0.06  1.72 | .01 | -.15  -0.01  0.73 | .14 | .27*  0.07  0.11  3.44* | .18 |
| **Caudate**  Std. β ^a^  R²  F  ΔR²  ΔF | .20  0.10  3.99* | -.34** | -.07  0.10  0.04  1.12 | -.18 | .17  0.04  2.17 | -.25 | -.19  0.09  0.08  2.56 | -.21 |
| **Putamen**  Std. β ^a^  R²  F  ΔR²  ΔF | -.11  0.03  1.88 | -.23 | -.08  0.01  0.01  0.34 | -.08 | -.09  < 0.01  1.10 | -.16 | -.03  -0.03  < 0.01  0.11 | -.06 |
| **Pallidum**  Std. β ^a^  R²  F  ΔR²  ΔF | .01  -0.01  0.83 | -.17 | .08  -0.04  0.01  0.21 | .04 | -.04  -0.01  0.76 | -.15 | .23  0.01  0.05  1.49 | -.01 |
| **Thalamus**  Std. β ^a^  R²  F  ΔR²  ΔF | -.33*  0.11  4.35* | -.21 | .07  0.09  0.02  0.55 | .12 | -.21  0.07  2.99 | -.27 | -.13  0.08  0.04  1.27 | .16 |
| **Lateral ventricle**  Std. β ^a^  R²  F  ΔR²  ΔF | .61**  0.40  21.71** | .31** | -.23*  0.47  0.09  4.59* | -.19* | .66**  0.43  22.06** | .33** | -.20*  0.51  0.09  5.31** | -.26* |
| Hierarchical multiple regression analyses for ET and PD patients. Regression analyses consisted of two steps: 1) demographic variables (age and sex) were entered as the first block, and 2) disease-related factors (duration of tremor and tremor severity measured by the CTRS) were entered in the second block.  ** p<0.01, * p<0.05  ^a^ all standardized regression coefficients are from the final step in the analyses.  Abbreviations: ET=Essential Tremor, DOT = Duration of tremor, CRST = Clinical Rating Scale for Tremor | | | | | | | | |

**Supplementary Table 7 Disease-related correlates of volumetric measurements in PD patients**

| **PD (n=29)** | | | | | | | | |
| --- | --- | --- | --- | --- | --- | --- | --- | --- |
|  | **Contralateral to more affected side** | | | | **Contralateral to less affected side** | | | |
|  | **Block 1** | | **Block 2** | | **Block 1** | | **Block 2** | |
|  | **Age** | **Sex** | **DOT** | **CTRS** | **Age** | **Sex** | **DOT** | **CTRS** |
| **Frontal lobe**  Std. β ^a^  R²  F  ΔR²  ΔF | -.15  -0.02  0.74** | .02 | -.27  -0.02  0.07  0.98 | .18 | -.18  -0.04  0.53 | .16 | -.28  0.05  0.15  0.19 | .35 |
| **Parietal lobe**  Std. β ^a^  R²  F  ΔR²  ΔF | -.15  -0.01  0.84 | -.17 | -.47*  0.16  0.22  0.36* | .29 | -.39  0.12  2.83 | -.14 | -.19  0.11  0.06  0.86 | -.14 |
| **Precuneus lobe**  Std. β ^a^  R²  F  ΔR²  ΔF | .07  -0.08  0.01 | -.10 | -.44  0.03  0.17  2.39 | .15 | -.03  -0.08  0.04 | -.06 | -.26  -0.08  0.08  0.95 | -.08 |
| **Occipital lobe**  Std. β ^a^  R²  F  ΔR²  ΔF | .06  -0.04  0.49 | .08 | -.54**  0.18  0.27  4.42* | .24 | -.05  0.15  3.37 | -.51 | -.25  0.15  0.06  0.97 | -.01 |
| **Temporal lobe**  Std. β ^a^  R²  F  ΔR²  ΔF | -.21  0.03  1.43 | -.31 | -.04  -0.02  0.03  0.44 | < .01 | -.29  0.01  1.07 | .02 | -.33  0.04  0.10  1.40 | .07 |
| **Hippocampus**  Std. β ^a^  R²  F  ΔR²  ΔF | -.23  -0.04  0.50 | -.03 | .04  -0.12  0.01  0.09 | -.09 | -.18  -0.04  0.47 | -.17 | -.43  0.09  0.19  2.77 | -.04 |
| **Parahippocampus**  Std. β ^a^  R²  F  ΔR²  ΔF | .07  -0.07  0.18 | .14 | .24  -0.10  0.05  0.64 | -.06 | -.02  -0.08  0.05 | .05 | .07  -0.10  0.06  0.78 | .25 |
| **Entorhinal lobe**  Std. β ^a^  R²  F  ΔR²  ΔF | -.49  0.08  2.19 | -.12 | .10  0.08  0.07  1.03 | -.30 | -.34  0.06  1.82 | -.13 | -.27  0.08  0.09  1.24 | -.09 |
| **Caudate**  Std. β ^a^  R²  F  ΔR²  ΔF | -.01  -0.07  0.09 | .09 | .18  -0.11  0.05  0.56 | .10 | .29  < 0.01  1.00 | .16 | .05  -0.07  0.02  0.19 | -.13 |
| **Putamen**  Std. β ^a^  R²  F  ΔR²  ΔF | .31  -0.03  0.58 | .20 | .26  0.18  0.25  4.14* | .44* | -.20  -0.07  0.09 | .02 | .24  0.27  0.37  6.90** | .57** |
| **Pallidum**  Std. β ^a^  R²  F  ΔR²  ΔF | -.30  0.10  2.48 | .18 | .14  0.08  0.05  0.68 | .15 | -.38  0.10  2.41 | .07 | .08  0.02  0.01  0.09 | -.04 |
| **Thalamus**  Std. β ^a^  R²  F  ΔR²  ΔF | -.08  -0.04  0.55 | -.17 | -.22  -0.02  0.09  1.18 | .29 | -.17  0.05  1.73 | -.30 | < .01  0.05  0.07  1.00 | -.28 |
| **Lateral ventricle**  Std. β ^a^  R²  F  ΔR²  ΔF | .70**  0.43  11.15** | .20 | .24  0.45  0.06  1.45 | .02 | .62**  0.34  7.88** | .29 | .31  0.39  0.09  2.05 | -.01 |
| Hierarchical multiple regression analyses for ET and PD patients. Regression analyses consisted of two steps: 1) demographic variables (age and sex) were entered as the first block, and 2) disease-related factors (duration of tremor and tremor severity measured by the CTRS) were entered in the second block.  ** p<0.01, * p<0.05  ^a^ all standardized regression coefficients are from the final step in the analyses.  Abbreviations: PD=Parkinson’s Disease, DOT = Duration of tremor, CRST = Clinical Rating Scale for Tremor | | | | | | | | |
